# Beyond a universal obesity microbiome signature: pre-intervention heterogeneity and a framework for baseline profiling

**DOI:** 10.3389/fnut.2026.1921813

**Published:** 2026-09-15

**Authors:** Penghui Liu, Runlin Mao, Na Li, Jiwu Guo, Jizhen Wang, Jie Mao

**Affiliations:** 1 The General Surgery Department, Lanzhou University Second Hospital, Lanzhou, China; 2 Marianapolis Preparatory School, Thompson, CT, United States

**Keywords:** baseline profiling, gut microbiota, metabolic phenotype, methodological heterogeneity, obesity, pre-intervention heterogeneity

## Abstract

Unlike previous reviews that primarily summarize obesity-associated microbial alterations, this review reframes the recurrent inconsistency of human obesity microbiome findings as an informative consequence of pre-intervention heterogeneity rather than merely failed replication or analytical noise. We integrate biological and contextual determinants of baseline microbiota variation with methodological and analytical sources of heterogeneity into a unified framework for interpreting why microbial diversity, taxonomic composition, and functional signals differ across individuals, populations, and studies. We critically synthesize evidence on baseline microbial diversity, community structure, host characteristics, regional dietary exposure, metabolic heterogeneity, and the potential relevance of pre-intervention microbiota to subsequent treatment outcomes. We also examine how cohort definition, stool sampling, laboratory processing, bioinformatic reconstruction, and statistical analysis shape the microbiome profiles that are ultimately observed. On this basis, we propose a baseline heterogeneity framework built on three linked principles: obesity-associated microbial signals are context-dependent; observed microbiota profiles are method- and pipeline-dependent; and their interpretation must be temporally anchored to the pre-intervention state. This framework positions baseline microbiota profiling not as descriptive cataloging or a search for a universal obesity-specific signature, but as a prerequisite for identifying confounding and effect modification, improving cross-population interpretation, and establishing the microbial and host context from which intervention begins. Future studies should integrate microbiota data with comprehensive characterization of relevant biological and contextual domains and evaluate the robustness of findings across analytical choices and independent populations. This conceptual shift provides a more rigorous foundation for region-specific baseline profiling and future longitudinal, mechanistic, and precision obesity research.

## 1 Introduction

Obesity is a major global health challenge associated with type 2 diabetes mellitus, cardiovascular disease, metabolic dysfunction-associated steatotic liver disease, several cancers, and increased mortality. Importantly, obesity is not a uniform condition defined solely by body mass index (BMI). Individuals with similar levels of adiposity may differ substantially in fat distribution, metabolic dysfunction, inflammatory status, organ impairment, and clinical risk (1–3). The gut microbiota provides a biologically plausible interface between environmental exposure and obesity-related metabolism through microbial energy harvest, short-chain fatty acid production, bile acid signaling, intestinal barrier function, inflammation, and insulin sensitivity (4–6). Yet this biological plausibility has not translated into a reproducible microbial signature of obesity in human populations.

For clarity, throughout this review, the term “microbiota” is used as shorthand for the gut microbiota, with the evidence synthesized here focused predominantly on bacterial communities; fungal and viral components of the intestinal ecosystem fall outside the present scope and are considered briefly in the Future Directions section (7–9). In this review, the pre-intervention baseline microbiota refers to the gut microbial community characterized before initiation of a planned dietary, behavioral, pharmacological, or surgical weight-loss intervention and before major intervention-related changes in host physiology or exposure. Self-initiated short-term changes in diet or physical activity and unsupervised use of over-the-counter weight-management products, probiotics, or prebiotics do not necessarily constitute a systematic intervention for this definition, but they remain potentially important microbiota-modifying exposures and should be documented with respect to type, intensity, duration, and timing before stool sampling and considered in stratified, adjusted, or sensitivity analyses when relevant. Recent body-weight trajectory and any history of intentional weight loss or weight regain should likewise be recorded, because a participant who is currently weight stable may not necessarily represent the same biological baseline as an individual with no recent or substantial history of weight loss. Accordingly, “pre-intervention” should be interpreted as a temporally defined and adequately characterized state rather than simply the absence of enrollment in a formal weight-loss program. Early obesity microbiome research sought relatively simple taxonomic markers, including phylum-level shifts and the Firmicutes-to-Bacteroidetes ratio (10). Subsequent reanalyzes (11, 12) and systematic reviews (13, 14), however, demonstrated substantial variation in microbial diversity, community composition, and obesity-associated taxa across studies. This inconsistency is biologically and methodologically plausible. Habitual diet, physical activity, smoking, sleep patterns, medication exposure, intestinal transit, and other host or environmental factors explain measurable microbiota variation and may confound apparently disease-associated signals (15–17). Their influence may also differ across populations; for example, staple food type, ethnicity, urbanization, and behavioral patterns have been associated with gut microbial structure in Chinese cohorts (18, 19). Metabolic heterogeneity provides an additional layer, because individuals with similar BMI may differ markedly in insulin resistance, dyslipidemia, fatty liver disease, and systemic inflammation, while differences in adiposity distribution may further modify metabolic risk (20).

These observations suggest that the central unresolved question is not simply which microorganisms are associated with obesity, but why reported associations differ across individuals, populations, and studies. Baseline microbiota features may also be associated with subsequent variation in weight-loss response, although current evidence remains largely observational or exploratory and does not establish clinically validated prediction (21). Previous reviews and systematic syntheses have extensively summarized obesity-associated taxa, microbial mechanisms, and comparisons between individuals with obesity and lean or non-obese controls (6, 13, 14). What remains insufficiently integrated is how these biological and contextual domains, together with methodological and analytical procedures, collectively generate the heterogeneity observed before intervention. For consistency, biological and contextual sources of pre-intervention heterogeneity are organized throughout this review into seven domains: host demographic factors, adiposity and body-fat distribution, regional dietary and environmental context, behavioral factors, medication and recent microbiota-modifying exposures, intestinal transit and stool characteristics, and metabolic phenotype. Methodological and analytical sources are considered separately.

Accordingly, this review treats inconsistency across human obesity microbiome studies as a scientific phenomenon requiring explanation rather than simply as failed replication or analytical noise. We propose a baseline heterogeneity framework organized around three linked principles: obesity-associated microbiota signals are context-dependent; observed microbiota profiles are method- and pipeline-dependent; and their interpretation must be temporally anchored to the pre-intervention state. Using this framework, we critically synthesize the biological, clinical, environmental, and methodological origins of baseline microbiota heterogeneity and consider their implications for region-specific profiling and future longitudinal and intervention research. Baseline characterization is therefore positioned not as descriptive cataloging or a search for a universal obesity-specific signature, but as a foundation for more reproducible cross-population interpretation and for understanding the microbial and host context from which intervention begins.

## 2 Review methodology

This narrative review was informed primarily by literature searches conducted in PubMed/MEDLINE, covering publications available from database inception to 23 August 2026. Broad search concepts combined terms related to obesity, overweight, or adiposity with terms related to the gut microbiota or gut microbiome. Topic-specific searches were used as needed to identify literature relevant to the principal themes of the review, including baseline or pre-intervention microbiota, microbial diversity and composition, host and demographic factors, diet and geographic context, medication and behavioral exposures, intestinal transit, metabolic phenotype, weight-loss response, and methodological aspects of microbiome research. Additional targeted searches were undertaken during manuscript preparation and revision, and reference lists of relevant reviews and key primary studies were consulted when useful for identifying additional pertinent literature.

Literature was assembled narratively according to its relevance to the conceptual framework and its contribution to interpreting biological, contextual, clinical, or methodological sources of heterogeneity. We generally prioritized peer-reviewed human studies directly relevant to obesity or overweight, together with large population-based studies, systematic reviews and meta-analyses, controlled interventions, and key methodological studies. Evidence from broader populations or preclinical studies was included selectively when it provided directly relevant contextual or mechanistic information not adequately addressed in obesity-specific human studies. Publications with limited relevance, insufficient methodological or outcome information, duplicate reporting without additional informative analyses, or non-peer-reviewed reporting were generally not emphasized. Representative studies were selected for detailed discussion on the basis of their relevance and informativeness, with particular attention to larger or methodologically informative studies, seminal work, and studies illustrating reproducible, null, inconsistent, or context-dependent findings. These considerations were used as general guides for the narrative synthesis rather than as prospectively prespecified eligibility criteria. Accordingly, no exhaustive systematic screening, formal risk-of-bias assessment, PRISMA-based study-selection procedure, or quantitative evidence synthesis was undertaken.

## 3 Biological context for interpreting microbiota heterogeneity in obesity

The gut microbiota may contribute to obesity-related metabolic dysfunction through several interconnected pathways. Microbial fermentation of dietary substrates generates short-chain fatty acids that participate in energy metabolism, appetite regulation, glucose homeostasis, intestinal barrier maintenance, and immune signaling (6, 22). Gut microorganisms also modify the composition of the bile acid pool, thereby influencing metabolic signaling through bile acid receptors involved in glucose regulation, lipid metabolism, energy expenditure, and inflammation (6, 23). In addition, impaired intestinal barrier integrity and increased exposure to microbial products may promote low-grade inflammation and insulin resistance (5, 24).

These mechanisms establish biological plausibility but do not imply that obesity should be associated with a single taxonomic or functional microbiota profile. The contribution of microbial fermentation, bile acid transformation, and inflammatory signaling depends on the substrates available from habitual diet and on host characteristics such as adiposity distribution, insulin sensitivity, liver function, intestinal transit, medication exposure, and baseline inflammatory status. Consequently, similar levels of adiposity may coexist with different microbial communities and metabolic outputs, whereas similar microbial features may have different physiological implications across dietary and metabolic contexts.

Controlled preclinical models provide an important complement to observational human microbiome studies. Germ-free and gnotobiotic animals, antibiotic-depletion models, defined microbial consortia, and fecal microbiota transplantation allow investigators to standardize host genetics, diet, housing, and environmental exposure while manipulating specific microbial communities or functions. These models can help establish temporal relationships, test candidate mechanisms, and assess whether particular microbial communities, taxa, or metabolites are sufficient or necessary to influence adiposity, insulin sensitivity, inflammation, and other metabolic outcomes. For example, transplantation of microbiota from human twin donors discordant for obesity into germ-free mice produced donor-dependent differences in adiposity and metabolic phenotype under controlled dietary conditions (25).

Nevertheless, translation from animal models to human obesity requires caution. Murine and human hosts differ in gastrointestinal anatomy, bile acid metabolism, immune and endocrine physiology, dietary exposure, and native microbial ecology (26). Germ-free animals also display atypical immune maturation and metabolic physiology compared with conventionally colonized mice (27, 28). Moreover, colonization with human rather than host-specific murine microbiota may not fully reproduce normal murine immune development, and human microbial communities may undergo incomplete or selective engraftment after transfer into mice (27–29). Recipient diet can further reshape the composition and functional configuration of transplanted human microbial communities (30). Thus, donor microbiota, recipient host context, and experimental conditions may all influence the microbial configuration and phenotype observed in gnotobiotic models (26, 29, 30).

In free-living human populations, variation across these biological and contextual domains cannot be standardized to the same extent and may confound or modify microbiota–metabolic associations (15–17). Consequently, preclinical models can establish mechanistic plausibility under controlled conditions, but they cannot by themselves determine the magnitude, consistency, or transportability of these effects in heterogeneous human populations. Their findings should therefore be triangulated with prospective human cohorts, controlled interventions, and direct functional or metabolite measurements before clinical inference.

Therefore, the principal relevance of these biological pathways to the present review is not that they define a universal obesity-associated microbiota, but that their expression is conditional on the host and environmental context. Pre-intervention microbiota profiles should accordingly be interpreted as integrated baseline states shaped by interactions among these biological and contextual domains rather than by adiposity alone. These context-dependent interactions are summarized in Figure 1.

**Figure 1:**
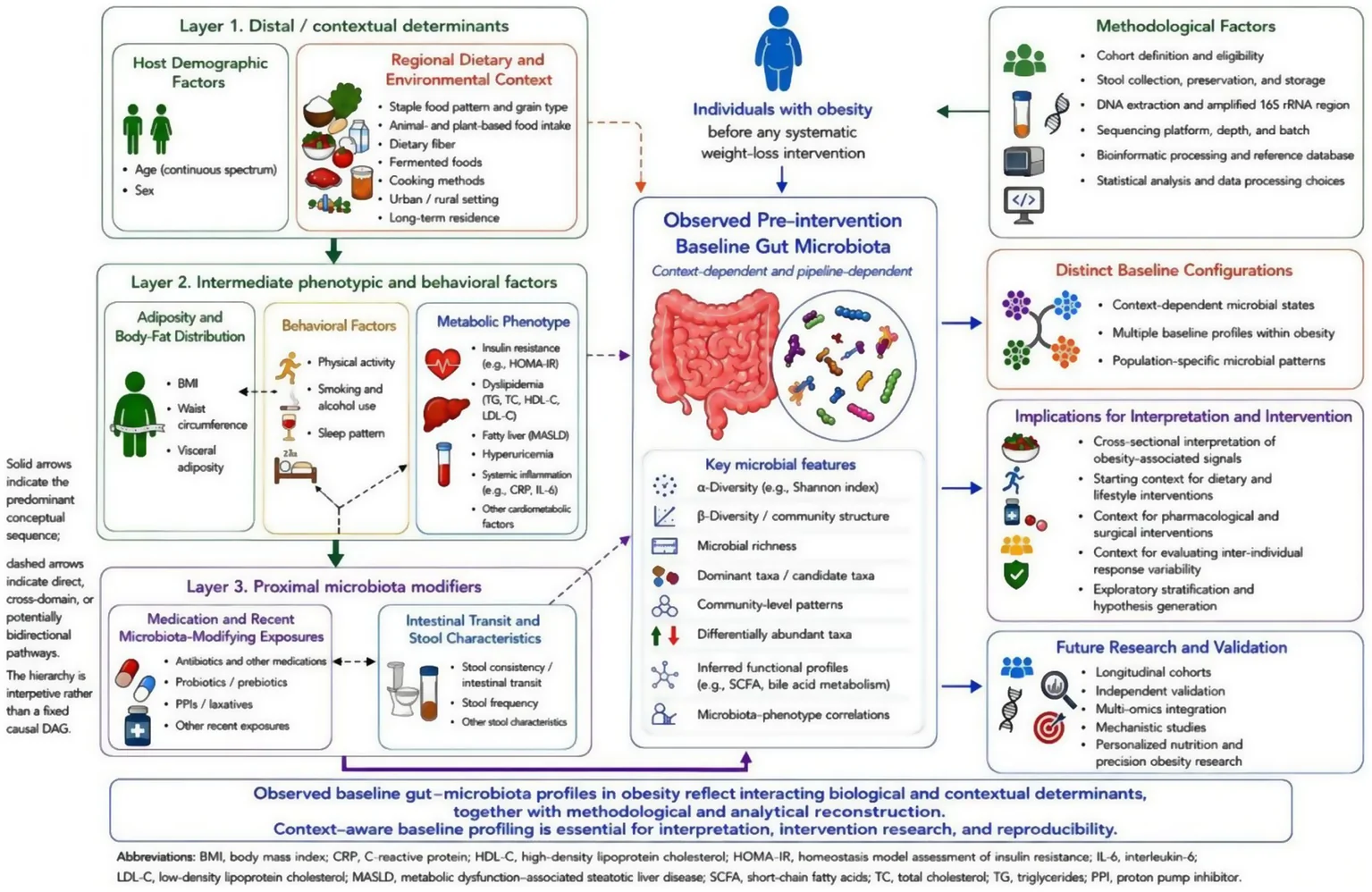
Hierarchical and integrative framework of pre-intervention baseline gut microbiota heterogeneity in obesity. The seven biological and contextual domains are organized along a heuristic distal–intermediate–proximal continuum rather than as mutually independent or causally equivalent determinants. Host demographic factors and regional dietary and environmental context generally occupy more distal positions; adiposity and body-fat distribution, behavioral factors, and metabolic phenotype represent intermediate layers; and medication/recent microbiota-modifying exposures together with intestinal transit and stool characteristics frequently act as more proximal modifiers. Solid arrows indicate the predominant conceptual sequence, whereas dashed arrows indicate direct, cross-domain, or potentially bidirectional pathways. The hierarchy is interpretive rather than a fixed causal DAG, and methodological and analytical factors influence how the underlying biological microbiota configuration is observed and reconstructed.

## 4 Baseline gut microbiota characteristics in individuals with obesity

Most evidence on obesity-associated gut microbiota characteristics has been derived from cross-sectional comparisons between individuals with obesity and lean or non-obese controls. These studies have reported differences in microbial diversity, community composition, taxonomic abundance, and functional potential, but the direction and magnitude of the findings have not been consistent across populations or across diversity metrics. Importantly, several study-level differences may help explain these contrasting observations. Peters et al. (31) analyzed 599 older American adults from two independent cohorts using 16S rRNA gene sequencing and BMI-based categories of healthy weight, overweight, and obesity. Obesity was associated with lower microbial richness, whereas pairwise differences in Shannon diversity and evenness were not statistically significant. In contrast, Thingholm et al. (32) analyzed 1,280 participants from two Northern German cohorts and explicitly separated lean non-diabetic individuals, obese individuals without type 2 diabetes, and obese individuals with type 2 diabetes. In this more metabolically stratified population, phylogenetic α-diversity was markedly lower in obese non-diabetic individuals than in lean participants.

Differences in sample size and analytical strategy are also important. Sze and Schloss (11) reanalyzed 10 independent 16S rRNA datasets using a largely harmonized analytical pipeline and found that significant α-diversity differences were detectable in only a minority of individual cohorts; pooled differences in richness, evenness, and Shannon diversity were statistically significant but small, and most individual studies lacked adequate power to detect modest diversity differences. At a broader evidence level, a systematic review and meta-analysis including 32 cross-sectional studies and 13,186 stool samples found lower Shannon diversity in individuals with obesity in only 9 of 22 studies reporting this metric. Only seven studies provided sufficiently comparable data for quantitative pooling, which yielded no significant overall difference and substantial between-study heterogeneity (13). The included studies also differed in population characteristics, obesity definitions, sequencing approaches, amplified regions, reference databases, and control of potential confounders. Taken together, these comparisons suggest that divergent α-diversity findings may reflect not only biological heterogeneity between populations, but also differences in metabolic stratification, sample size and statistical power, obesity classification, diversity metrics, sequencing and bioinformatic procedures, and covariate control.

Population-scale studies provide an important quantitative context for these inconsistencies. In the Flemish Gut Flora Project, BMI was significantly associated with overall microbiota composition but had only a small non-redundant effect, whereas stool consistency emerged as the strongest single microbiome covariate; the 18 non-redundant host and exposure variables retained in the multivariable model collectively explained 7.63% of community variation (16). In the LifeLines-DEEP metagenomic cohort, 126 intrinsic and exogenous factors together explained 18.7% of inter-individual variation in microbial composition, with most individual factors contributing only modest effects (15). These findings indicate that BMI, as a proxy for adiposity, is a comparatively weak determinant of community-level gut microbiota variation rather than a dominant organizing factor. Consequently, an obesity-associated microbial signal may be readily attenuated or altered by variation in diet, medication exposure, intestinal transit, metabolic phenotype, and other correlated host characteristics. Apparent “obesity-associated” microbial signatures should therefore be interpreted as potentially reflecting a mixture of adiposity-related effects and correlated biological or contextual influences rather than as microbiota markers attributable to adiposity alone.

Baseline heterogeneity may influence the interpretation of obesity-associated microbiota findings through several distinct but overlapping mechanisms. First, habitual dietary patterns, medication exposure, stool characteristics, physical activity, smoking, and sleep patterns may confound obesity–microbiota associations because these factors may be associated with both obesity status and microbial composition. In this context, habitual dietary patterns encompass both the variety of foods and food groups consumed and the relative intake of major dietary components, including macronutrients, dietary fiber, and specific food sources. Stool consistency should be interpreted primarily as a readily accessible proxy for colonic transit time rather than as an isolated fecal characteristic. Slower transit generally permits greater colonic water reabsorption and prolongs the residence time of substrates and microorganisms, whereas faster transit is commonly associated with looser stools and more rapid luminal clearance. These differences can influence substrate availability, microbial growth dynamics, community richness, and overall microbiota composition. However, Bristol Stool Scale scores provide only an indirect estimate of transit and should not be considered equivalent to direct transit-time measurement. Stool consistency should therefore be recorded and considered in covariate adjustment or sensitivity analyses, while direct assessment of intestinal transit may be warranted when transit-related mechanisms are central to the research question (33). Large population-based studies have shown that stool consistency, diet, medication exposure, and other host or environmental factors explain measurable variation in gut microbiota composition and diversity (15, 16). Disease-oriented analyses have further demonstrated that inadequate control of host variables can generate apparently disease-specific microbial signals (17). Second, factors such as age, sex, geographic background, and dietary pattern may modify the direction or magnitude of microbiota associations, thereby limiting their transportability across populations. Third, metabolic phenotype may capture microbiota-associated variation that is not represented by BMI alone. This does not imply greater causal proximity; rather, it reflects the possibility that metabolic characteristics such as insulin resistance, dyslipidemia, and systemic inflammation may be associated with differences in microbial diversity or composition among individuals who are all overweight or obese. For example, Kim et al. (20) reported lower α-diversity and distinct gut microbial composition in metabolically unhealthy compared with metabolically healthy individuals within a cohort of 747 adults with overweight or obesity. Consistent with this observation, lower microbial gene richness has been associated with greater adiposity, insulin resistance, dyslipidemia, and systemic inflammation (34). These findings support considering metabolic phenotype alongside BMI when interpreting obesity-associated microbiota signals, while not establishing that metabolic abnormalities are causally closer to microbial variation. These mechanisms may coexist within the same cohort and should therefore be distinguished during study design, statistical adjustment, and interpretation. As illustrated in Figure 1, these determinants do not act independently but converge to generate multiple baseline microbial configurations within individuals with obesity.

Microbial richness has received particular attention as a potentially relevant marker of metabolic heterogeneity. However, richness captures only the number of observed or estimated taxa or microbial genes and does not reflect their relative distribution or phylogenetic relationships. It is also sensitive to sequencing depth, feature-detection thresholds, the contribution of rare taxa, and bioinformatic processing. The observation that richness differed whereas Shannon diversity and evenness did not differ significantly within the same cohort further illustrates that conclusions may depend on the selected metric (31). Richness should therefore be regarded as one dimension of α-diversity rather than as a preferred or comprehensive measure.

Commonly used α-diversity metrics capture related but non-equivalent dimensions of microbial community structure. Richness-sensitive measures emphasize the number of detected features, whereas Shannon diversity additionally incorporates evenness, Simpson-based measures are more strongly influenced by dominant taxa, and Faith’s phylogenetic diversity incorporates evolutionary breadth. Consequently, the same biological contrast may appear stronger, weaker, or even absent depending on the metric selected, particularly when group differences are concentrated in rare taxa, community evenness, dominance structure, or phylogenetic composition. Studies should therefore prespecify complementary α-diversity metrics and report effect estimates and uncertainty rather than treating α-diversity as a single interchangeable construct. Discordant results across metrics should be interpreted as potentially reflecting different dimensions of community structure rather than selectively emphasizing the index that reaches statistical significance. Accordingly, α-diversity should be treated as a multidimensional baseline descriptor rather than as a standalone definition of obesity-associated dysbiosis.

At the taxonomic level, human studies have reported obesity-associated differences involving genera such as *Bacteroides*, *Prevotella*, *Faecalibacterium*, *Roseburia*, *Blautia*, *Ruminococcus*, *Akkermansia*, *Bifidobacterium*, *Oscillibacter*, and *Alistipes*. However, the direction and magnitude of these associations have varied considerably across cohorts (31, 32, 35). *Akkermansia* provides an illustrative example of why taxon-level associations should be interpreted in relation to host metabolic context rather than adiposity alone. In overweight and obese adults, Dao et al. (36) found no significant difference in *Akkermansia muciniphila* abundance between overweight and obese groups, whereas higher baseline abundance was associated with lower fasting glucose, a lower waist-to-hip ratio, smaller subcutaneous adipocytes, and a generally healthier metabolic profile. Higher baseline abundance was also associated with greater improvement in several metabolic parameters during calorie restriction (36). These findings support an association between *A. muciniphila* abundance and metabolic phenotype within individuals with excess body weight, but they do not establish that dietary, geographic, medication-related, or transit-related factors modify the association between *A. muciniphila* and adiposity.

In addition, genus-level 16S rRNA profiles may combine species or strains with different biological properties, while differences in sequencing and analytical procedures may affect whether and how a taxon is detected. Failure to reproduce the same direction or magnitude of association therefore does not necessarily indicate that one study is biologically incorrect; it may reflect effect modification, residual confounding, limited taxonomic resolution, or methodological reconstruction. A candidate taxon should be considered robust only when its association persists after adjustment for major covariates, remains stable across reasonable analytical approaches, and is replicated in independent populations, ideally with functional or metabolite-level support.

The consequences of baseline heterogeneity become clearer when mapped onto the microbiome research pipeline. At the cohort-definition stage, participant selection, definitions of obesity and metabolic health, comparator composition, habitual diet, medication exposure, intestinal transit, and metabolic comorbidity determine the biological contrast under study. These factors may alter both baseline community structure and the apparent magnitude or direction of obesity-associated effects (15–17). Accordingly, an observed difference in β-diversity or in a specific taxon may reflect unequal biological or contextual characteristics between cohorts rather than adiposity itself. Conversely, failure to reproduce an association in another cohort may arise from several non-mutually exclusive explanations, including genuine population-specific effects, differences in sample size or statistical power, variation in obesity or comparator definitions, unequal covariate distributions, and differences in sampling, sequencing, or analytical procedures. Non-replication should therefore be interpreted cautiously and should not, by itself, be taken as evidence either for limited cross-population transportability or for the absence of a biological relationship.

At the pre-analytical and laboratory stages, sample collection and preservation, DNA extraction, the amplified 16S rRNA region, sequencing depth, and batch structure can introduce systematic shifts in observed relative abundances and taxonomic resolution (37–39). These effects are particularly consequential when technical batches are aligned with clinical or metabolic subgroups, because technical and biological variation then become difficult to separate. Standardized procedures and randomization across extraction and sequencing batches can reduce within-study bias, but they do not make taxon-level findings automatically comparable across studies using different protocols.

At the analytical stage, the selected α-diversity index, β-diversity distance metric, feature-filtering threshold, taxonomic database, compositional method, covariate set, and multiple-testing strategy influence which microbial signals are detected and how strongly they appear to be associated with obesity (37, 38). β-Diversity is useful for characterizing overall community-level heterogeneity, but apparent group separation should be interpreted together with dispersion and batch structure because PERMANOVA results may be influenced by differences in within-group variability (40). Likewise, taxon-level associations that weaken or change direction after adjustment for diet, medication exposure, stool consistency, or metabolic phenotype should not be presented as obesity-specific. Functional profiles inferred from 16S rRNA data introduce an additional layer of uncertainty because they estimate potential functions on the basis of taxonomic composition rather than directly measuring microbial genes, transcripts, proteins, or metabolites (41).

The Firmicutes-to-Bacteroidetes ratio provides a historical illustration of the limitations of reducing obesity-associated microbiota variation to a single phylum-level measure. Although an early study reported obesity-associated shifts in these bacterial phyla (10), subsequent reanalyzes and focused evaluations showed that the direction and magnitude of the ratio vary substantially across populations and analytical settings (11, 12). Systematic evidence similarly indicates that this ratio does not consistently distinguish individuals with obesity across cohorts (13, 14). Contemporary obesity microbiome research should therefore prioritize community-level structure, complementary α-diversity metrics, finer-resolution taxa, functional potential, and integration with host metabolic phenotypes rather than reliance on a single taxonomic ratio.

The relative impact of different sources of heterogeneity is context-dependent rather than fixed. When sample collection and laboratory procedures are uniform and technical batches are balanced across groups, biological and clinical heterogeneity and incomplete covariate control are likely to have the greatest influence on interpretation. However, technical procedures can themselves produce substantial shifts in observed microbiota profiles. For example, Costea et al. (39) compared 21 DNA extraction protocols applied to the same fecal samples and found that DNA extraction was the largest technical contributor to variation in metagenomic community composition, with measurable effects on diversity estimates and taxonomic representation. If such protocol- or batch-related variation is systematically aligned with the exposure or clinical group, technical and biological effects may become difficult to distinguish. Analytical choices further determine which dimension of the microbial community is emphasized and how robust an association appears, but they cannot compensate for poorly defined cohorts, unbalanced confounders, or incomplete metadata.

The most credible microbiome findings are therefore those that remain stable across complementary diversity metrics and reasonable analytical choices, are robust to covariate adjustment and sensitivity analyses, and are reproducible across independent populations or supported by direct functional measurements. Baseline microbiota profiles in obesity should consequently be interpreted as context-dependent and pipeline-dependent estimates rather than fixed disease-defining signatures. Their principal value in first-stage cross-sectional studies lies in characterizing internal heterogeneity, identifying candidate microbial patterns, and generating testable hypotheses for subsequent longitudinal and interventional research.

## 5 Age, sex, and interacting host determinants of baseline microbiota heterogeneity

Age is a major host determinant of gut microbiota composition and should be carefully considered in obesity microbiome research. Across adulthood and aging, the gut microbiota may change in relation to diet, immune function, medication exposure, physical activity, hormonal status, gastrointestinal physiology, and metabolic health. Large-scale human studies have identified age-related microbial trajectories across different populations, and recent Chinese data further suggest that age is an important covariate affecting gut microbiota composition (42, 43). These findings support careful adjustment for age when evaluating microbiota–metabolic associations, because age may be associated with both microbial composition and metabolic characteristics. However, current evidence does not establish that age systematically modifies the associations between gut microbiota and specific metabolic traits in obesity; such effect modification would require formal interaction testing or adequately powered age-stratified analyses.

For adult obesity cohorts, age should generally be retained as a continuous variable in primary analyses, with flexible modeling considered when a nonlinear relationship is plausible. This recommendation is supported by microbiome studies showing that the apparent age–microbiota association can depend on how age is modeled. de la Cuesta-Zuluaga et al. (44) compared simple linear regression with linear spline models in adult cohorts from the United States, the United Kingdom, Colombia, and China. In three of the four populations, spline models provided a better fit for the association between age and α-diversity, revealing an increase in microbial diversity during younger adulthood followed by a plateau after approximately 40–45 years (44). By contrast, categorical approaches may be informative when age groups represent prespecified biological or life-stage distinctions; for example, Wu et al. (43) compared five age groups spanning childhood to older adulthood and identified differences in α-diversity, β-diversity, and taxonomic composition across these groups. These examples indicate that neither continuous nor categorical modeling is universally optimal. Continuous age, potentially modeled with nonlinear terms, is preferable when the objective is to preserve information and characterize gradual trajectories, whereas categorical analyses should be reserved for biologically or empirically justified age ranges or exploratory presentation.

Sex is another host factor that may contribute to baseline microbiota heterogeneity in individuals with obesity. Sex-associated microbial differences have been reported across adult populations, although their magnitude and taxonomic direction are not fully consistent (42, 45). Importantly, some obesity-associated microbial patterns appear to differ by sex. In a cohort of 551 Chinese adults, Gao et al. (46) found that the BMI-associated pattern of α-diversity remained evident among women but was not significant across BMI categories among men; obesity-associated taxonomic differences also differed between women and men. These findings illustrate that the apparent association between adiposity and microbiota may depend on cohort sex composition, although they do not establish that sex imbalance itself is a general cause of between-study inconsistency.

Age and sex should not be interpreted as isolated determinants of gut microbiota composition because both are correlated with other biological and contextual characteristics that may also influence the microbiota. Empirical evidence supports age-dependent variation in some sex-associated microbial patterns. For example, Zhang et al. (42) reported an age-related decrease in sex-dependent differences in gut microbiota composition across several adult populations. Similarly, de la Cuesta-Zuluaga et al. (44) found that sex differences in α-diversity were more pronounced in younger than in middle-aged adults; however, formal age-by-sex interaction terms were not significant after correction for multiple comparisons. These findings indicate that age-stratified differences may occur but should not be interpreted as evidence of universal age-by-sex effect modification. More generally, correlated host characteristics can confound microbiota associations (17), but current evidence does not establish that age- or sex-associated microbial signals consistently weaken after adjustment for such variables. Studies should therefore account for major correlated covariates and formally test prespecified interaction terms when age- or sex-dependent effect modification is hypothesized.

The interpretation of age and sex is particularly important in obesity because adiposity distribution and metabolic phenotype vary substantially across demographic groups. Individuals with similar BMI may differ in visceral fat accumulation, insulin resistance, dyslipidemia, fatty liver disease, inflammatory activity, and cardiometabolic risk, and the distribution of these characteristics may vary by age and sex. Metagenomic evidence from Chinese adults further indicates that age, sex, and BMI are each associated with taxonomic and functional microbiome composition (47). These overlapping associations highlight the potential for demographic confounding but should not be interpreted as evidence of age- or sex-dependent modification of specific microbiota–metabolic relationships unless such interactions are formally tested.

For pre-intervention microbiota profiling in obesity, age and sex should be treated as host demographic factors and analyzed alongside the other biological and contextual domains defined above. Age should preferably be modeled as a continuous variable, with flexible nonlinear terms considered where appropriate; categorical analyses should be used when age ranges are biologically or empirically justified or for exploratory presentation. Differences in cohort age structure and sex composition should also be considered when interpreting apparently discordant microbiota findings across studies, particularly when age- or sex-specific patterns are suggested by the data. Formal interaction or stratified analyses may help distinguish effect modification from confounding when adequately supported, but such findings should be interpreted cautiously rather than assumed to represent universal demographic effects.

## 6 Regional dietary and environmental context and community-level microbial patterns

Geography and habitual diet are among the strongest contextual determinants of inter-individual variation in the human gut microbiota. In this review, regional dietary and environmental context encompasses habitual dietary exposure together with the broader regional setting in which that exposure occurs. Its components include geographic background, long-term residence, urban–rural setting, cultural food practices, staple-food structure, food availability, cooking practices, and relevant socioeconomic context. Behavioral factors are treated as a separate domain and refer primarily to physical activity, smoking, sleep patterns, alcohol use, and other habitual behaviors. For consistency, alcohol use is classified under behavioral factors in the present framework.

Large population-based studies have shown that microbial composition is associated with diet, medication exposure, stool characteristics, behavioral factors, and geographic background (15–17). In Chinese populations, staple food type, ethnicity, urbanization, and behavioral factors have also been associated with gut microbial structure (18, 19). These findings are particularly relevant to obesity research because regional dietary context is linked not only to microbial composition, but also to adiposity, insulin resistance, lipid metabolism, fatty liver disease, systemic inflammation, and other metabolic traits. It should therefore be considered a key contextual factor when interpreting baseline microbiota profiles in individuals with obesity.

Although historically influential, the enterotype concept provides only a limited descriptive framework for diet-associated microbial variation. Early metagenomic studies proposed that gut microbial communities could be grouped into configurations characterized predominantly by *Bacteroides*, *Prevotella*, or *Ruminococcus*, and subsequent work associated *Bacteroides*- and *Prevotella*-enriched profiles with different long-term dietary patterns (48, 49). However, these configurations should not be interpreted as discrete, stable, or universally reproducible biological categories. Gut microbial communities frequently vary along continuous compositional gradients, and clustering results depend on cohort composition, sequencing strategy, taxonomic resolution, distance metrics, and clustering methods. Enterotype assignment should therefore be regarded as an exploratory analytical approach rather than a primary classification framework or clinical endpoint.

Dietary exposure may influence gut microbiota over both long-term and short-term time scales. Long-term dietary patterns can shape relatively stable microbial configurations, whereas short-term dietary changes may rapidly alter microbial community structure and microbial gene expression (50). Cross-population dietary studies have also shown that traditional high-fiber diets and industrialized Western-style diets are associated with markedly different microbial patterns (51). In obesity, these dietary effects are especially important because regional food structure, staple grain type, meat intake, dairy consumption, vegetable and fruit intake, dietary fiber, fermented foods, alcohol consumption, and cooking methods may all influence both baseline microbiota and metabolic phenotype. As a result, comparisons of obesity-associated microbiota across regions should not be interpreted without considering dietary background.

Dietary exposure is itself a major source of methodological heterogeneity across microbiome studies. Food frequency questionnaires are generally designed to characterize habitual intake over an extended period and are practical for large cohorts, but their estimates depend on the food list, reference period, portion-size assumptions, participant recall, and the cultural relevance of the questionnaire. By contrast, 24 h dietary recalls and food records or diaries provide greater temporal and food-level detail, but a single or limited number of assessment days may not adequately represent usual intake because of day-to-day and seasonal variation. Repeated administrations can improve the estimation of habitual intake (52). Dietary records collected close to stool sampling may also better capture short-term exposures that are temporally relevant to microbial composition.

Dietary assessment methods may influence microbiome interpretation through differences in exposure windows, timing relative to stool collection, food-composition databases, and the way dietary data are summarized. Studies may characterize diet using individual nutrients, food groups, dietary patterns, or composite indices, but these representations capture related rather than equivalent dimensions of exposure. Different dietary indices derived from the same food frequency questionnaire may explain different proportions of gut microbiota variation (53). High-frequency paired dietary and stool sampling has further shown that daily microbial variation may reflect dietary intake over the preceding several days and may be more closely associated with specific food choices than with conventional nutrient summaries (54). These methodological differences may attenuate true diet–microbiota associations, produce residual dietary confounding, or generate apparent between-cohort and regional differences that partly reflect exposure measurement rather than biological variation.

Beyond differences in dietary assessment methods, inadequate control of total energy intake and macronutrient composition represents an important source of confounding in obesity microbiota research. Total energy intake, the relative contributions of fat, carbohydrate, and protein, and dietary fiber can each influence the substrates available to intestinal microorganisms and thereby affect community composition and microbial metabolic activity. Controlled dietary studies demonstrate that substantial microbiota changes can occur after short-term shifts between animal- and plant-based diets (50), and that differences in dietary fat and carbohydrate composition can alter microbial taxa and fecal metabolites even under isocaloric feeding conditions (55). Because dietary intake is also closely related to adiposity and metabolic phenotype, microbiota differences observed between individuals with and without obesity may therefore partly reflect correlated dietary exposures rather than adiposity alone. Accordingly, studies examining obesity–microbiota associations should, when dietary data of sufficient quality are available, consider total energy intake together with major macronutrient composition and dietary fiber in study design and multivariable analysis. Associations derived without adequate assessment or adjustment of these dietary factors should be interpreted cautiously because residual dietary confounding may contribute to apparently obesity-associated microbial signals.

Future studies should therefore report the dietary assessment instrument, reference period, number and timing of administrations, cultural adaptation and validation, food-composition database, and the temporal relationship between dietary assessment and stool sampling. For pre-intervention baseline profiling, a validated population-specific food frequency questionnaire may be suitable for characterizing habitual diet, whereas repeated 24 h recalls or short-term food records collected around stool sampling can provide complementary information on recent exposure. When feasible, findings should be examined using both food-based and nutrient- or pattern-based representations rather than assuming that one dietary summary fully captures the microbial effects of diet.

Taxon-dominance patterns require particularly cautious interpretation. Although *Prevotella* enrichment has frequently been associated with carbohydrate- or fiber-rich diets, *Prevotella* is not a biologically uniform genus. Different species, strains, and clades may have distinct ecological and metabolic implications. For example, the *Prevotella copri* complex comprises multiple genetically distinct clades whose distribution and associations with host health may vary across populations (56). This limitation is particularly relevant to 16S rRNA sequencing, which frequently lacks species- or strain-level resolution. *Prevotella*-enriched profiles should therefore be interpreted as population- and diet-associated community signals rather than as fixed enterotypes or direct markers of metabolic health or disease.

For region-specific obesity microbiota research, the principal objective should be to contextualize baseline microbial heterogeneity rather than to claim regional uniqueness prematurely. In regions with distinctive dietary patterns, variables such as staple food structure, grain type, meat and dairy consumption, vegetable and fruit intake, fermented foods, alcohol use, and urban–rural living context may help explain baseline microbiota variation. However, without a local normal-weight control group or direct cross-regional comparisons using harmonized sampling, sequencing, and analytical protocols, it would be inappropriate to conclude that the microbiota of individuals with obesity from a particular region is definitively distinct from that of other populations. A more scientifically appropriate objective is to establish a regionally contextualized baseline microbiota profile, characterize internal heterogeneity, and compare the observed patterns cautiously with published findings from other geographic and dietary settings.

Overall, regional dietary and environmental context should be interpreted through continuous and population-dependent patterns rather than fixed enterotype categories. It should also be considered in relation to the other biological and contextual domains defined in this review rather than as an isolated determinant of microbiota variation. This integrated approach may help distinguish microbial variation associated with adiposity from variation driven by long-term diet, regionally patterned behaviors, exposure measurement, and population structure.

## 7 Metabolic phenotypes and microbiota heterogeneity within obesity

Obesity is metabolically heterogeneous. In this review, metabolic phenotype refers to the integrated metabolic state of an individual, characterized primarily by glucose–insulin homeostasis, lipid metabolism, hepatic metabolic health, urate metabolism, and systemic inflammatory and cardiometabolic status. It therefore encompasses measures such as fasting glucose, fasting insulin, insulin resistance, circulating lipid profiles, metabolic dysfunction-associated steatotic liver disease and related liver indicators, serum uric acid, and inflammatory markers. Metabolic phenotype is distinguished from demographic characteristics such as age and sex and from adiposity itself; however, adiposity distribution, particularly visceral adiposity, is closely related to metabolic risk and may interact with these metabolic characteristics.

Individuals with similar BMI may therefore differ substantially in insulin resistance, dyslipidemia, fatty liver disease, hyperuricemia, systemic inflammation, and overall cardiometabolic risk. This heterogeneity is central to the interpretation of gut microbiota findings because microbial patterns may be associated not only with adiposity itself, but also with the metabolic abnormalities that accompany obesity (3, 20, 57). Therefore, microbiota studies that classify participants only by BMI may miss clinically meaningful variation within the obesity population.

Metabolically healthy obesity (MHO) and metabolically unhealthy obesity (MUO) provide a useful framework for examining metabolic heterogeneity within obesity, but there is no universally accepted definition of either phenotype. Studies differ in the number and type of metabolic abnormalities permitted and in the thresholds applied to blood pressure, fasting glucose, triglycerides, HDL cholesterol, waist circumference, insulin resistance, and medication use (57). Consequently, the proportion of participants classified as MHO or MUO—and the microbial differences observed between these groups—may vary according to the operational criteria used.

MHO should therefore not be regarded as a benign, permanent, or biologically uniform condition. Rather, it describes relatively preserved metabolic homeostasis according to a specified set of criteria and at a particular time point (57). In a large study of adults with overweight or obesity, participants classified as metabolically unhealthy showed lower microbial α-diversity and distinct gut microbiota composition compared with those classified as metabolically healthy. Specific taxa, including *Oscillospira* and Coriobacteriaceae, were associated with a more favorable metabolic profile (20). These findings support the relevance of metabolic stratification, but comparisons across studies should account for differences in MHO/MUO definitions. Future microbiome studies should report the exact classification criteria and, when feasible, conduct sensitivity analyses using alternative definitions rather than treating MHO and MUO as universally standardized categories.

Insulin resistance is particularly relevant in obesity-related microbiota research. Gut microbial functions involved in carbohydrate utilization, short-chain fatty acid production, bile acid metabolism, amino acid metabolism, and inflammatory signaling may contribute to impaired insulin sensitivity (6, 22, 23). Recent multi-omics evidence has shown that fecal carbohydrates, particularly host-accessible monosaccharides, are increased in individuals with insulin resistance and are associated with microbial carbohydrate metabolism and host inflammatory signatures (58). These findings support considering fasting glucose, fasting insulin, and HOMA-IR when insulin resistance is relevant to the study question. They also suggest that microbial functional potential may be associated with metabolic dysfunction in ways that are not captured by adiposity alone.

Metabolic dysfunction-associated steatotic liver disease and systemic inflammation also deserve attention. Most earlier microbiome studies used the term non-alcoholic fatty liver disease, but the underlying clinical concept is closely related to obesity, insulin resistance, lipid metabolism, bile acid signaling, and the gut–liver axis (6, 23, 59). Gut microbiota alterations have been associated with fatty liver severity and shifts in microbial metabolic function, supporting the relevance of liver-related variables in obesity microbiome research (60). Low-grade inflammation, often reflected by C-reactive protein or other inflammatory markers, may be related to altered gut barrier function, microbial metabolites, metabolic endotoxemia, and adipose tissue inflammation (5, 24). Fatty liver status, liver enzymes, lipid-related indicators, and inflammatory markers may therefore provide relevant metabolic context when these phenotypes are pertinent to the specific microbiota–phenotype relationship under investigation.

Serum uric acid may be considered an additional exploratory metabolic variable. Hyperuricemia is common in individuals with obesity and may reflect dietary purine or fructose exposure, renal urate handling, insulin resistance, and systemic metabolic dysfunction. Emerging evidence suggests that the gut microbiota may participate in urate homeostasis through purine degradation, intestinal urate handling, inflammatory regulation, and gut barrier function (61). However, evidence from obesity-specific cohorts remains limited. Associations between uric acid and gut microbiota should therefore be interpreted cautiously and framed as hypothesis-generating rather than causal.

Overall, the most informative approach for baseline obesity microbiota research is not limited to comparing individuals with obesity against non-obese controls. Instead, it should characterize microbiota heterogeneity within obesity in relation to both adiposity and metabolic phenotype. Depending on the research question, adiposity measures such as BMI and waist circumference may be evaluated together with selected metabolic indicators, including measures of glucose–insulin homeostasis, lipid metabolism, hepatic metabolic health, urate metabolism, and systemic inflammation (57, 61). Importantly, these variables may serve different analytical roles—as confounders, mediators, effect modifiers, outcomes, or correlated phenotypes—depending on the hypothesized relationship. They should therefore be considered according to the specific research question rather than routinely entered together into every adjusted model. This framework is particularly suitable for exploratory cross-sectional studies that aim to establish a pre-intervention microbiota profile and generate hypotheses for future longitudinal research. The major biological and contextual determinants of pre-intervention gut microbiota heterogeneity in obesity are summarized in Table 1, together with a qualitative assessment of the strength and limitations of the current evidence.

**Table 1:** Major determinants of pre-intervention gut microbiota heterogeneity in obesity and the strength of current evidence.

Determinant	Representative variables	Potential microbiota relevance	Evidence strength	Interpretation and current limitations
Age	Chronological age; aging-related changes in diet, immunity, physical activity, medication exposure, and gastrointestinal physiology	May influence α-diversity, β-diversity, dominant taxa, and microbial functional potential	Moderate	Large population studies support age-related microbiota variation, but associations vary across populations and may be confounded by diet, medication use, frailty, and metabolic health. Age should preferably be modeled continuously, with cohort-specific categories used cautiously. Age-associated patterns may partly operate through these correlated factors rather than through an independent pathway (42–44)
Sex	Biological sex; hormonal status; adiposity distribution; immune regulation; bile acid metabolism; dietary behavior	May contribute to differences in microbial composition, metabolic pathways, immune-associated patterns, and microbiota–phenotype relationships	Limited to moderate	Sex-associated microbiota patterns have been reported but are not consistently reproduced across populations. Some obesity-associated patterns differ by sex, although evidence that sex imbalance itself explains between-study inconsistency remains limited. Sex effects may overlap with hormonal status, adiposity distribution, behavior, and metabolic phenotype (42, 45, 47)
Adiposity and body-fat distribution	BMI; waist circumference; visceral adiposity; overall and central obesity; recent weight trajectory; history of intentional weight loss or weight regain	May be associated with microbial richness, community structure, energy-related pathways, and microbiota–metabolic interactions	Moderate	Associations with BMI alone are inconsistent, and population-level analyses suggest that BMI contributes only modestly to overall microbiota variation. Adiposity distribution may be more informative than overall body size, although direct evidence remains limited. Adiposity overlaps strongly with metabolic phenotype, and recent weight trajectory and history of weight loss or regain should be considered when defining the baseline state (3, 15, 16, 20, 34)
Regional dietary and environmental context	Long-term residence; urban or rural setting; cultural dietary practices; staple-food pattern; grain type; food availability; cooking methods; animal- and plant-based food intake; dietary fiber; fermented foods	May shape community-level compositional gradients, taxon-dominance patterns, microbial diversity, and carbohydrate fermentation capacity	Moderate to strong	Diet and geographic context are consistently associated with gut microbiota variation, but their obesity-specific contribution is difficult to separate from ethnicity, socioeconomic conditions, urbanization, medication use, and methodological differences. This domain includes both distal contextual factors and habitual dietary exposures that may directly influence the microbiota (18, 19, 51)
Behavioral factors	Physical activity; smoking; sleep pattern; alcohol use; other long-term habitual behaviors	May influence microbial diversity, community structure, inflammatory tone, and microbiota–host metabolic interactions	Limited to moderate	Associations are biologically plausible but exposure measurement is heterogeneous and residual confounding is common. Obesity-specific causal evidence remains limited, and behavioral effects may operate directly or indirectly through diet, adiposity, metabolic status, and intestinal transit (15–17)
Medication and recent exposures	Antibiotics; probiotics; prebiotics; proton pump inhibitors; metformin; lipid-lowering drugs; laxatives; recent infection; gastrointestinal disturbance	May substantially alter microbial composition, richness, specific taxa, and inferred functional profiles	Strong	Antibiotics and several commonly used medications are established determinants of gut microbiota variation. Effects depend on drug class, dose, duration, recency, and host context. Medication effects may overlap with intestinal transit and metabolic phenotype, and confounding by indication should be considered (15–17)
Stool consistency and intestinal transit	Bristol Stool Scale; bowel frequency; directly measured transit time when available	May influence microbial growth rates, richness, community structure, and relative taxonomic abundance	Moderate to strong	Stool consistency is consistently associated with microbiota composition and is a practical but imperfect proxy for colonic transit. Transit may be influenced by diet, medications, host physiology, and metabolic state and can therefore act as both a mediator and a proximal modifier (33)
Metabolic phenotype	Fasting glucose; fasting insulin; HOMA-IR; lipid profile; fatty liver or MASLD; liver enzymes; serum uric acid; CRP and other inflammatory markers	May be associated with microbial diversity, carbohydrate and bile acid metabolism, gut barrier function, inflammation-related pathways, and microbiota–phenotype correlations	Moderate	Microbiota differences have been reported across metabolic phenotypes, but findings depend on phenotype definitions and may reflect correlated diet, medication use, adiposity, and liver disease. Evidence is stronger for insulin resistance and fatty liver than for hyperuricemia or individual inflammatory markers. Metabolic phenotype overlaps substantially with adiposity and may act as an intermediate phenotype (20, 58, 60, 61)

BMI, body mass index; CRP, C-reactive protein; HOMA-IR, homeostasis model assessment of insulin resistance; MASLD, metabolic dysfunction-associated steatotic liver disease. Evidence grading: Evidence strength was qualitatively classified according to the consistency of findings across studies, the number and scale of supporting human cohorts, the directness of evidence for pre-intervention microbiota heterogeneity in obesity, and susceptibility to residual confounding. Strong indicates broadly consistent evidence from multiple human studies; moderate indicates generally supportive but heterogeneous or partly indirect evidence; and limited indicates emerging, inconsistent, or predominantly exploratory evidence. The determinant categories are organizational rather than mutually exclusive; their causal position may vary across research questions, and cross-domain mediation, confounding, interaction, and feedback should be expected. Intermediate categories indicate evidence falling between the two adjacent levels. These categories do not represent a formal GRADE assessment or establish causality.

## 8 Pre-intervention baseline microbiota and clinical relevance

Studying the gut microbiota before weight-loss intervention has specific clinical relevance because baseline microbial communities may reflect long-term host–diet–environment interactions. Unlike post-intervention microbiota profiles, which are influenced by dietary restriction, medication exposure, surgery, weight loss, altered gastrointestinal physiology, and metabolic improvement, pre-intervention microbiota profiles capture the microbial state before major external modification. Therefore, baseline profiling may help clarify why individuals with obesity differ in metabolic phenotype and provide a reference for evaluating intervention response (21).

Evidence from dietary and lifestyle interventions suggests that baseline gut microbiota features may be associated with inter-individual variation in weight-loss response, although the endpoints and analytical approaches differ across studies. In a 16-week calorie-restriction study, Dong et al. (62) reported that a random-forest classifier based on baseline 16S rRNA profiles discriminated participants who subsequently achieved at least 5% weight loss, with an internally cross-validated area under the receiver operating characteristic curve of 0.80. However, only 46 participants completed the study, 14 achieved the predefined weight-loss threshold, and the model was not validated in an independent cohort. In a PREVIEW sub-study of 211 adults with overweight and prediabetes, baseline microbiota features explained approximately 25% of the variation in total body-fat change during an 8-week low-energy diet (63). Diener et al. (64) identified 31 baseline metagenomic functional features and bacterial replication rates associated with subsequent weight loss after adjustment for age, sex, and baseline BMI; however, the metagenomic analysis was conducted in a selected subcohort of 25 individuals with extreme weight-change phenotypes. Cuevas-Sierra et al. (65) developed sex- and diet-specific models combining baseline microbiota and genetic scores in 190 Spanish adults assigned to moderately high-protein or low-fat hypocaloric diets, but these models were developed within the same study population and require independent validation.

Taken together, these studies provide preliminary and hypothesis-generating evidence rather than clinically established predictive evidence. Their strengths include prospective dietary interventions, microbiota assessment before treatment, and the use of clinically relevant weight or body-composition outcomes. However, the overall evidence remains limited by modest effective sample sizes, exploratory or internally developed prediction models, selected extreme-response subgroups, heterogeneous interventions and endpoints, differences in follow-up duration, sequencing strategies, and analytical pipelines, and potential residual confounding from baseline adiposity, habitual diet, medication use, physical activity, metabolic health, and intervention adherence. Most reported microbial signatures have not undergone robust external validation across independent ethnic, dietary, and technical settings, and their calibration, incremental predictive value beyond conventional clinical variables, and clinical decision-making utility remain insufficiently established.

From a causal-inference perspective, the current evidence should be interpreted in graded terms. Although several studies have measured the gut microbiota prospectively before weight-loss intervention, the baseline microbial exposure itself has not been experimentally assigned and remains correlated with habitual diet, adiposity, metabolic phenotype, medication use, physical activity, and subsequent intervention adherence. These studies therefore support baseline microbiota features primarily as candidate response-associated or prognostic biomarkers rather than as established causal determinants of weight-loss efficacy. Dietary restriction and weight loss can themselves reshape the gut microbiota (66), further complicating causal interpretation. A higher level of evidence would require direct experimental perturbation of the microbiota; however, a multicenter randomized trial of lean-donor fecal microbiota transplantation in individuals with severe obesity did not improve either presurgical or postsurgical weight loss compared with autologous transplantation (67). Thus, current human evidence does not establish specific baseline microbial features as causal intervention targets. Such a role would require convergent support from controlled microbiota-directed interventions, prospective longitudinal studies with repeated sampling and careful assessment of adherence and confounding, and complementary causal-inference approaches. Until such evidence is available, baseline microbiota features should be described as candidate response-associated biomarkers or internally predictive features rather than as causal targets or clinically validated predictors.

Baseline microbiota profiling may also be relevant to surgical and pharmacological weight-loss strategies, although the evidence base is less mature than that for dietary intervention. Bariatric surgery can induce durable changes in gut microbial composition, microbial metabolites, bile acid-related pathways, and host metabolic outcomes (68, 69). Nevertheless, whether preoperative microbiota can reliably predict surgical weight-loss response, diabetes remission, or long-term weight regain remains unresolved. Similarly, for pharmacological weight-loss therapies, future studies should determine whether baseline microbiota modifies treatment response or whether observed microbiota changes mainly reflect weight loss, altered diet, or improved metabolic status. These questions require prospective designs with baseline and serial stool sampling, detailed dietary and medication records, and standardized clinical endpoints.

For first-stage exploratory cross-sectional studies, the objective should not be to prove treatment prediction. If participants are sampled only before intervention, the appropriate aim is to describe baseline microbiota characteristics and explore their associations with the seven biological and contextual domains defined above. Prediction of intervention response should be considered a subsequent longitudinal research question requiring follow-up clinical outcomes and repeated microbiota sampling.

Accordingly, a baseline cross-sectional study in individuals with obesity before any systematic weight-loss intervention can establish a pre-intervention microbiota profile, identify major sources of internal heterogeneity, and generate hypotheses for prospective cohorts. Such a study should avoid overclaiming causality or predictive value. Its main contribution lies in defining the microbial, host, dietary, and metabolic context from which future dietary, lifestyle, pharmacological, or surgical weight-loss interventions begin.

## 9 Methodological sources of heterogeneity in 16S rRNA-based obesity microbiome studies

16S rRNA gene sequencing remains a practical approach for first-stage taxonomic profiling because it is relatively accessible and can characterize α-diversity, β-diversity, overall community structure, and taxonomic composition in moderately sized clinical cohorts. However, a 16S-derived microbiota profile is not a direct or method-independent representation of the underlying microbial community. It is shaped by successive decisions involving cohort definition, sampling, laboratory processing, bioinformatic reconstruction, and statistical analysis. These sources of methodological heterogeneity do not contribute equally in every study, and their influence depends largely on whether they are randomly distributed across participants or systematically aligned with clinical groups (37, 38).

Methodological heterogeneity begins before stool collection. Inclusion and exclusion criteria determine whether the intended comparison reflects adiposity itself or a mixture of adiposity, metabolic dysfunction, medication exposure, recent dietary change, and previous intervention. Sampling after antibiotic exposure, bowel preparation, probiotic or prebiotic supplementation, major dietary modification, or initiation of microbiota-modifying medication may produce a profile that no longer represents the intended pre-intervention state (37, 38). In obesity cohorts, incomplete information on habitual diet, medication use, intestinal transit, adiposity distribution, insulin resistance, fatty liver disease, and inflammatory status may be more consequential than many downstream technical differences because these variables influence both microbial composition and the biological contrast under investigation (15–17). Once such metadata have not been collected, laboratory standardization and statistical adjustment cannot fully reconstruct the missing biological context.

Dietary exposure illustrates how measurement choices can generate residual heterogeneity even when the same covariate is nominally recorded. Food frequency questionnaires, 24 h recalls, and food records capture different exposure periods and differ in their sensitivity to habitual intake, recent food consumption, recall error, and day-to-day variation. The timing of dietary assessment relative to stool sampling and the representation of diet as nutrients, food groups, dietary patterns, or composite indices may therefore affect the estimated diet–microbiota association. Consequently, adjustment for a broadly defined “dietary pattern” does not necessarily ensure equivalent control of dietary confounding across cohorts.

Stool collection and handling can introduce further variation through differences in the interval between defecation and stabilization, collection devices, preservation methods, transport conditions, storage temperature, and freeze–thaw exposure (37–39). These factors are most problematic when handling procedures differ systematically between comparison groups or study sites. Uniform handling reduces within-study bias, but it does not guarantee direct comparability with cohorts using different preservation and storage protocols. Stool consistency requires separate consideration because it is partly a proxy for intestinal transit time and therefore represents biological variation rather than merely a technical characteristic of the specimen. Failure to distinguish transit-related variation from handling-related variation may lead investigators either to overlook an important confounder or to treat a genuine host characteristic as a laboratory artifact.

Among laboratory procedures, DNA extraction and selection of the amplified 16S rRNA region are particularly influential for taxon-level comparability. Extraction protocols differ in their efficiency for disrupting bacterial cell walls, while primer pairs vary in taxonomic coverage and amplification bias. As a result, the relative abundance or apparent absence of specific taxa may differ across studies even when the underlying microbial communities are similar (37–39). Sequencing depth mainly affects the recovery of rare features and richness-sensitive outcomes when coverage is inadequate, whereas extraction and primer-related biases can systematically alter the representation of particular microbial groups. Negative controls and mock communities improve quality assessment, but they cannot completely harmonize findings generated using fundamentally different extraction or amplification strategies.

Batch structure may become one of the most influential technical sources of heterogeneity when it is confounded with clinical grouping. If samples from one obesity or metabolic subgroup are predominantly extracted or sequenced in one batch and samples from another subgroup in a different batch, biological and technical variation become difficult to separate. Randomizing clinical groups across extraction and sequencing batches is therefore more informative than merely recording batch identifiers after processing. Statistical batch adjustment may reduce residual variation, but it cannot reliably correct a design in which batch and exposure group are nearly or completely aligned (37, 38, 70).

Bioinformatic processing determines which sequence features are retained and how they are represented. Denoising procedures, ASV or OTU construction, filtering thresholds, reference databases, and taxonomic assignment algorithms may produce different feature tables and taxonomic profiles (71–73). These choices tend to exert the greatest influence on low-abundance features and fine taxonomic resolution, whereas strong community-level patterns may be more stable across reasonable pipelines. Nevertheless, agreement at the phylum or genus level should not be interpreted as equivalence at the species, strain, or functional level. Transparent reporting of software versions, reference databases, filtering criteria, and feature-construction methods is therefore necessary to determine whether discordant findings reflect biological differences or analytical reconstruction.

Statistical choices can alter not only statistical significance but also the direction and apparent specificity of microbiota associations. Microbiome data are compositional, meaning that an apparent increase in the relative abundance of one taxon may result from decreases in other community members rather than from absolute microbial expansion (74).

This limitation is particularly relevant to taxon-level claims of microbial “enrichment” or “depletion.” Relative abundance alone cannot distinguish true expansion or loss of a taxon from proportional redistribution caused by changes in other community members or in total microbial biomass. Quantitative microbiome profiling has demonstrated that correcting for microbial load can materially alter biological interpretation; for example, an apparent Bacteroides–Prevotella trade-off observed in relative profiles was no longer significant after conversion to quantitative profiles (75). Recent obesity-specific data further illustrate the importance of this distinction. In adolescents with obesity, phylum-level relative composition appeared broadly similar across many participants, whereas flow-cytometry-calibrated profiling revealed marked variation in absolute fecal bacterial biomass, ranging from 3.8 × 10^9 to 5.5 × 10^11 bacteria per gram of dry stool, with microbial biomass associated with stool consistency, diversity, and metabolic and inflammatory features (76). Accordingly, taxon-level differences derived solely from relative abundance should not be interpreted as evidence of absolute microbial expansion or depletion. When feasible, sequencing-based relative profiles should be complemented by measurements of total microbial load or taxon-specific abundance using approaches such as flow cytometry, quantitative PCR, or validated internal-standard strategies; when such measurements are unavailable, conclusions should explicitly remain framed in relative-abundance terms.

Diversity metrics are also not interchangeable. Richness-based measures, Shannon diversity, Simpson diversity, and Faith’s phylogenetic diversity capture different ecological dimensions, and discordant results across these indices should not be reduced to a single generalized change in α-diversity. For β-diversity, PERMANOVA findings should be interpreted together with dispersion assessment because apparent group separation may reflect differences in within-group variability rather than differences in group centroids (40).

Covariate selection is similarly critical for taxon-level analysis. Associations that weaken or change direction after adjustment for age, sex, BMI, habitual diet, medication exposure, stool consistency, adiposity distribution, or metabolic phenotype should not be interpreted as robust obesity-specific signals. Compositional bias-corrected models and multivariable association frameworks may improve analytical robustness (77, 78), but they cannot compensate for inadequate sample size, incomplete metadata, severe group imbalance, or excessive analytical flexibility. The use of several plausible pipelines without prespecified priorities may also increase the risk of selectively emphasizing the analysis that produces the most favorable result.

Functional inference from 16S rRNA data introduces an additional layer of uncertainty. Tools such as PICRUSt2 estimate potential pathways from taxonomic composition and reference-genome information rather than directly measuring microbial genes, transcription, proteins, or metabolites (41). Variation in taxonomic resolution and reference-genome representation may therefore propagate into apparently different functional profiles. Inferred pathways should be regarded as exploratory hypotheses, particularly for strain-dependent functions or pathways that are poorly represented in reference databases. Direct functional conclusions require validation using shotgun metagenomics, metatranscriptomics, metabolomics, bile acid profiling, short-chain fatty acid measurement, or other complementary approaches.

The relative importance of these sources of heterogeneity is context-dependent rather than fixed. When stool handling, laboratory procedures, and technical batches are standardized and balanced across groups, cohort definition, host heterogeneity, metadata completeness, and covariate control are likely to exert the greatest influence on biological interpretation. Conversely, when sample processing or sequencing batches are aligned with clinical groups, technical and biological effects may become difficult to distinguish. DNA extraction and amplified-region selection are particularly important for cross-study taxon-level comparability, whereas inadequate sequencing depth disproportionately affects rare-feature and richness analyses. Bioinformatic and statistical choices determine which dimensions of the community are emphasized and how robust an association appears, but they cannot rescue poorly defined biological contrasts or severely confounded study designs.

Complete control or standardization of all biological and contextual exposures is rarely feasible in real-world human cohorts. The practical objective should therefore be to standardize measurement and sample processing rather than to eliminate naturally occurring exposure variation. Variables should be prioritized according to their expected effect on the microbiota, their relationship with the primary research question, and the feasibility and reliability of their measurement. Recent antibiotics, bowel preparation, acute gastrointestinal illness, major recent dietary change, and other strong microbiota-modifying exposures should receive the highest priority and may justify exclusion, delayed sampling, or dedicated sensitivity analyses. A minimum core dataset should include age, sex, adiposity measures, stool consistency, major microbiota-modifying medications, key metabolic phenotypes, and the timing and handling of stool samples. Detailed dietary intake, physical activity, sleep, socioeconomic conditions, and broader environmental exposures may then be collected as extended or context-specific variables, particularly when they are central to the study hypothesis. This tiered approach is more realistic than attempting exhaustive standardization and reduces the risk that limited resources are distributed across numerous weakly measured covariates while more influential sources of heterogeneity remain uncontrolled.

Comprehensive metadata reporting is also important for the reuse and cross-study interpretation of microbiome sequencing data. When sequencing data are deposited in public repositories, relevant sample-level metadata should be provided alongside them in a standardized and interpretable form, including key demographic and clinical characteristics, pre-sampling exposures, stool collection and handling information, and major laboratory and sequencing variables, where ethically and legally permissible. Consistent linkage of sequencing data with such metadata can facilitate cross-study comparisons, assessment of potential sources of heterogeneity, reproducible reanalysis, and future meta-analysis (38). Sensitive participant information should be shared only in appropriately de-identified or controlled-access forms.

Methodological robustness should therefore be evaluated according to whether findings remain stable across complementary diversity metrics, reasonable bioinformatic and statistical choices, covariate-adjusted models, and sensitivity analyses, and whether they are reproducible in independent cohorts or supported by directly measured functional data. The objective is not to eliminate all variability, which is unrealistic, but to distinguish biological heterogeneity from variation introduced by measurement and analysis. The major methodological sources of heterogeneity, their likely effects on microbiome findings, and their relative interpretive importance are summarized in Table 2.

**Table 2:** Major methodological sources of heterogeneity and their effects on 16S rRNA-based obesity microbiome findings.

Stage	Principal source of heterogeneity	Likely effect on microbiome findings	Relative impact and interpretation
Cohort definition and sampling time	Differences in obesity and metabolic-health definitions, eligibility criteria, comparator composition, recent weight trajectory, prior history of weight loss or regain, previous interventions, antibiotics, probiotics/prebiotics, bowel preparation, major dietary change, and microbiota-modifying medications	Alters the biological contrast under investigation and may produce non-comparable “baseline” populations	Often among the most influential sources when laboratory procedures are standardized. Missing information on pre-sampling exposures cannot be fully corrected analytically (37, 38)
Biological and contextual metadata	Incomplete or inconsistently measured variables across the seven biological and contextual domains, including total energy intake, macronutrient composition, and dietary fiber	Produces residual confounding and may cause dietary, host, or exposure-related microbial signals to be misattributed to obesity or adiposity	Adequate biological and contextual metadata are essential for interpretation. When diet is relevant to the analytical question, total energy intake, major macronutrients, and dietary fiber should be considered in covariate selection. Statistical adjustment cannot compensate for severely under-represented groups or exposures that were not measured or were measured imprecisely (15, 17, 50, 53, 55)
Dietary exposure assessment	Use of food frequency questionnaires, 24 h recalls, or food records with different reference periods, administration frequency, food-composition databases, and timing relative to stool sampling	Captures non-equivalent dimensions and time scales of diet, attenuating associations and limiting cross-cohort comparability	Particularly important when regional dietary differences are central to the study. A broadly defined dietary pattern may not adequately capture or control differences in total energy intake, macronutrient composition, and dietary fiber across cohorts (52–55)
Stool consistency and sample handling	Biological variation in intestinal transit together with differences in collection devices, delay to stabilization, preservation, transport, storage, and freeze–thaw exposure	Transit affects richness, microbial growth, and community composition, whereas handling differences may introduce technical shifts	Stool consistency should be treated primarily as a biological proxy for transit, not merely a technical variable. Handling effects are most problematic when procedures differ systematically by group or study site (33, 37–39)
DNA extraction, amplified region, and sequencing depth	Differences in cell-lysis efficiency, primer coverage, 16S rRNA region, sequencing platform, and read depth	Produces taxon-specific recovery, apparent absence of taxa, and variable detection of rare features	DNA extraction and amplified-region selection are major determinants of taxon-level comparability. Inadequate depth mainly affects richness and low-abundance features (37–39)
Quantification strategy	Reliance on relative abundance alone versus incorporation of total microbial load or absolute taxon quantification using flow cytometry, quantitative PCR, or internal-standard approaches	Relative enrichment or depletion may reflect changes in other community members or total microbial biomass rather than true expansion or loss of the taxon	Particularly important for taxon-level associations. Absolute or load-aware profiling can distinguish proportional redistribution from changes in microbial quantity, although each quantification method has its own measurement biases. When absolute measurements are unavailable, enrichment or depletion should be explicitly interpreted as relative abundance changes (74–76)
Batch structure and quality control	Alignment of extraction or sequencing batches with clinical groups; contamination; inconsistent use of negative controls and mock communities	May create, obscure, or exaggerate apparent group differences	Potentially dominant when batch and clinical group are confounded. Randomization across batches is more effective than *post hoc* statistical correction, which cannot fully repair severe design confounding (37, 38, 70)
Bioinformatic reconstruction	Differences in denoising, ASV or OTU construction, feature filtering, reference databases, taxonomic assignment, and software versions	Generates different feature tables and taxonomic profiles, particularly for low-abundance and fine-resolution taxa	Strong community-level patterns may be relatively stable, whereas species-level and rare-feature findings are more pipeline-dependent. Transparent reporting is essential for interpreting discordant results (71–73)
Statistical analysis and covariate control	Compositional treatment, choice of α- and β-diversity metrics, PERMANOVA without dispersion assessment, multiple testing, model specification, and incomplete covariate adjustment	Changes effect estimates, significance, and the apparent specificity of obesity-associated signals	Analytical methods can improve robustness but cannot rescue inadequate sample size, incomplete metadata, or poorly defined cohorts. Stability across reasonable models is more informative than a single significant result (40, 74, 78)
Outcome selection and functional inference	Selective emphasis on particular diversity indices, taxa, community clusters, or PICRUSt2-predicted pathways	Different indices capture different ecological dimensions, while inferred functions may appear more definitive than the underlying evidence supports	Complementary α-diversity metrics should be reported separately. Taxon-dominance patterns and 16S-based functional predictions are exploratory and require cautious interpretation or direct multi-omics validation (37, 38, 41)
Pragmatic implementation and variable prioritization	Limited feasibility of comprehensively standardizing or measuring diet, behavioral factors, medication, socioeconomic, and environmental exposures	Incomplete or imprecise measurement may leave residual confounding, while excessive data collection may reduce data quality and participant adherence	Prioritize strong microbiota-modifying exposures, core host and metabolic variables, stool consistency, and standardized sample processing. Collect extended domain-specific variables according to the research question and available resources; acknowledge variables that cannot be reliably controlled
Validation and interpretation	Lack of sensitivity analyses, independent replication, longitudinal sampling, or direct functional measurements	Limits reproducibility, transportability, and distinction between stable baseline features, metabolic correlates, and candidate predictors	The most credible findings are robust across analytical choices and covariate adjustment and are reproduced in independent cohorts or supported by direct functional data (62–64)

ASV, amplicon sequence variant; OTU, operational taxonomic unit; PERMANOVA, permutational multivariate analysis of variance; PICRUSt2, phylogenetic investigation of communities by reconstruction of unobserved states 2.

## 10 The baseline heterogeneity framework for context-aware microbiota profiling in obesity

We define the baseline heterogeneity framework as an interpretive model in which the pre-intervention gut microbiota observed in an obesity cohort is jointly shaped by two interconnected dimensions. The first comprises seven biological and contextual domains: host demographic factors, adiposity and body-fat distribution, regional dietary and environmental context, behavioral factors, medication and recent microbiota-modifying exposures, intestinal transit and stool characteristics, and metabolic phenotype. These domains are intended as an organizational taxonomy rather than as mutually exclusive or causally equivalent compartments. The second dimension comprises methodological and analytical reconstruction through cohort definition, sample collection and processing, sequencing, bioinformatic analysis, and statistical modeling. Together, these categories distinguish broad sources of biological and contextual heterogeneity from the procedures through which the underlying microbiota profile is observed, while recognizing that the causal position, temporal proximity, and relative contribution of individual determinants may differ across pathways.

Within the biological and contextual dimension, the seven domains should be interpreted along a heuristic distal–intermediate–proximal continuum rather than as parallel, mutually independent determinants or as a fixed linear causal chain. Host demographic factors and the broader regional and environmental context generally occupy more distal positions because they can shape downstream exposures and host physiology. Adiposity and body-fat distribution, behavioral factors, and metabolic phenotype commonly represent intermediate phenotypic or behavioral layers through which more distal influences may be expressed. Medication and recent microbiota-modifying exposures, together with intestinal transit and stool characteristics, often act as more proximal modifiers of the intestinal ecological environment. This ordering is not absolute. Habitual diet, although nested within regional dietary and environmental context, can also exert rapid effects on microbial composition (50), while medications may produce drug-specific microbiota changes, as demonstrated for metformin (79). Intestinal transit is itself closely associated with microbial community structure (33). Cross-domain overlap is therefore expected: medication exposure may influence the microbiota through multiple gastrointestinal and metabolic pathways, while adiposity, metabolic state, behavioral factors, and intestinal transit may be interrelated. Accordingly, the seven domains are best understood as interacting and partially overlapping causal layers rather than as independent predictors. The analytical role of any individual variable—as an exposure, mediator, confounder, effect modifier, or outcome—should therefore be specified according to the research question, temporal sequence, and hypothesized causal pathway.

Based on current evidence, region-specific obesity microbiota research should be designed to characterize baseline microbial heterogeneity across these seven biological and contextual domains. For a first-stage study, an exploratory cross-sectional design is practical and scientifically appropriate. Individuals with obesity can be enrolled before any systematic weight-loss intervention, and stool samples can be collected for 16S rRNA sequencing together with metadata representing each of these domains. Where resources permit, measurement of total microbial load may complement relative sequencing profiles, particularly for taxon-level interpretation. In this setting, baseline β-diversity may be considered a reasonable primary microbiota outcome because it reflects overall community-level structure, whereas complementary α-diversity metrics capturing richness, evenness, dominance, and phylogenetic breadth, together with dominant taxa, exploratory community-level or taxon-dominance patterns, differentially abundant taxa, inferred functional profiles, and microbiota–phenotype correlations, can be analyzed as secondary or exploratory outcomes (37, 40, 41).

The central research question should be framed as the characterization of pre-intervention microbiota features and sources of internal heterogeneity, rather than as definitive proof of regional uniqueness. This distinction is especially important for underrepresented regions or populations with distinctive dietary and behavioral patterns. Regional dietary context should be evaluated through long-term residence, urban or rural setting, cultural food practices, staple-food pattern, grain type, food availability, cooking methods, animal- and plant-based food intake, dietary fiber, fermented foods, alcohol consumption, and other habitual dietary characteristics. The dietary assessment method should also be prespecified and transparently reported, because food frequency questionnaires, 24 h dietary recalls, and food records capture different exposure periods and may not produce directly comparable estimates across populations. Previous Chinese population studies have shown that staple food type, ethnicity, urbanization, behavioral patterns, and dietary background are associated with gut microbial structure, supporting the need to incorporate regional dietary variables into obesity microbiota research (18, 19).

Metabolic phenotyping is an important component of baseline microbiota characterization in obesity, but the specific variables selected should depend on the research question and hypothesized microbiota–phenotype relationship. Beyond BMI, relevant measures may include waist circumference and selected indicators of glucose–insulin homeostasis, lipid metabolism, hepatic metabolic health, urate metabolism, and systemic inflammation. If participants are classified as metabolically healthy obesity or metabolically unhealthy obesity, the exact operational definition should be reported because no universally accepted criteria exist and classification may differ substantially across definitions (57). These variables may help distinguish microbial patterns associated primarily with adiposity from those associated with insulin resistance, dyslipidemia, fatty liver disease, hyperuricemia, or systemic inflammation (57, 61). This approach is particularly important because obesity defined by BMI alone may obscure clinically meaningful metabolic heterogeneity.

Age and sex should be considered as host demographic factors that may contribute to baseline microbiota heterogeneity and may also correlate with other biological and contextual characteristics. Age is generally most informative when retained as a continuous variable, with nonlinear or categorical representations used when biologically or empirically justified (42–44). Differences in cohort age structure and sex composition should be considered when comparing findings across studies, particularly when demographic-specific patterns are suggested by the data (42, 45, 47). Formal interaction or stratified analyses may help distinguish effect modification from confounding when adequately supported, but such analyses should not be interpreted as demonstrating universal age- or sex-specific effects.

Methodological standardization is essential for region-specific baseline profiling. For prospective baseline profiling, recent weight trajectory should be assessed together with major changes in diet, physical activity, medication use, and gastrointestinal health. A prespecified period of relative weight stability before stool sampling is preferable; for example, a change of no more than approximately 3% of body weight over the preceding 3 months may serve as a pragmatic study criterion, although the optimal threshold is not standardized and should be reported explicitly. Major recent dietary changes, initiation or substantial modification of microbiota-relevant medications or supplements, acute gastrointestinal illness, bowel preparation, and recent antibiotic exposure should prompt exclusion, delayed sampling, or sensitivity analysis according to the study objective and exposure intensity. Sample collection, preservation, transport, storage, DNA extraction, sequencing region, bioinformatic pipeline, diversity metrics, statistical models, and reporting standards should be prespecified and transparently described (37–39). Because microbiome data are compositional and highly susceptible to confounding, analyses should incorporate appropriate transformation or normalization, multiple-testing correction, covariate adjustment, and sensitivity analyses for major confounders such as medication exposure and stool consistency (40, 74, 78).

In real-world cohorts, however, complete standardization of host behavior and environmental exposure is neither feasible nor necessarily desirable, because such variation is part of the population context that baseline profiling aims to characterize. A pragmatic design should distinguish variables that require strict procedural standardization from variables that require careful measurement and analytical control. Stool collection, preservation, laboratory processing, and batch allocation should be standardized as closely as possible. By contrast, diet, physical activity, medication use, and environmental exposure should generally be documented rather than artificially homogenized. Data collection may be organized into a minimum core set and an extended context-specific set. The core set should prioritize age, sex, BMI and waist circumference, stool consistency, recent antibiotics and other major microbiota-modifying medications, key metabolic indicators, and major habitual dietary characteristics. More detailed dietary, behavioral, socioeconomic, and environmental measures can be added according to the study hypothesis, regional context, sample size, and available resources. Variables that cannot be measured reliably should be acknowledged as potential sources of residual confounding rather than treated as fully controlled.

A practical analytical framework may therefore include three levels. The first level describes the baseline microbial profile, including complementary α-diversity metrics, β-diversity and overall community structure, dominant taxa, and exploratory community-level patterns. The second level evaluates the seven biological and contextual domains defined above. The third level generates hypotheses for future longitudinal research by identifying baseline microbial features that may be associated with metabolic risk or potential intervention responsiveness. This stepwise framework helps avoid overinterpretation of cross-sectional data while preserving their value for future dietary, lifestyle, pharmacological, or surgical weight-loss studies (62, 66, 68).

The expected contribution of region-specific baseline microbiota research is not the discovery of a universal obesity-specific taxon. Rather, its value lies in establishing a regionally contextualized pre-intervention microbiota profile and identifying major sources of baseline heterogeneity among individuals with obesity. Such positioning is particularly suitable for first-stage exploratory studies in underrepresented populations. It avoids overclaiming causality, regional uniqueness, or treatment prediction, while providing a foundation for subsequent longitudinal cohorts, intervention studies, multi-omics analyses, and personalized metabolic risk stratification. A proposed workflow for region-specific baseline gut microbiota profiling in obesity is shown in Figure 2. Taken together, these observations support an integrative model of pre-intervention baseline gut microbiota heterogeneity in obesity. The seven biological and contextual domains do not operate independently or at a single causal level. Rather, distal and contextual determinants may influence baseline microbial configurations through intermediate phenotypic and behavioral factors and more proximal modifiers, while direct pathways, cross-domain interactions, and feedback relationships may coexist. At the same time, cohort definition, stool collection and preservation, laboratory procedures, bioinformatic processing, and statistical analysis influence how these underlying configurations are reconstructed and ultimately observed. Baseline microbiota profiles should therefore be interpreted as both context-dependent and pipeline-dependent. These profiles shape the interpretation of cross-sectional obesity-associated signals, define the microbial context from which dietary, lifestyle, pharmacological, or surgical interventions begin, and provide a basis for evaluating inter-individual response variability in future longitudinal studies. This integrative framework is illustrated in Figure 1.

**Figure 2:**
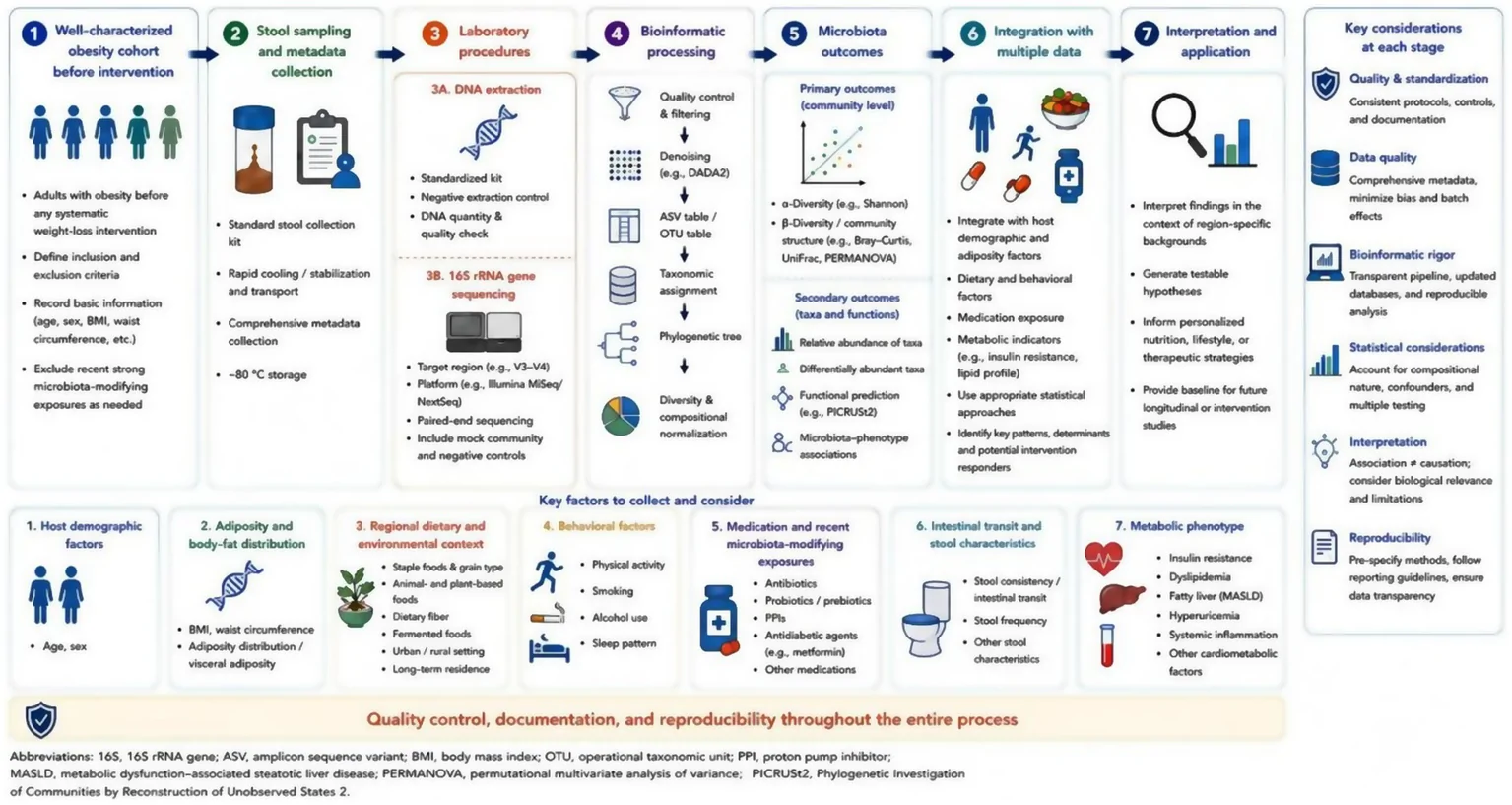
Proposed workflow for region-specific baseline gut microbiota profiling in obesity. The workflow includes pre-intervention cohort definition, standardized stool sampling, metadata collection, laboratory procedures, 16S rRNA gene sequencing, bioinformatic processing, microbiota outcome assessment, integration with host and metabolic data, and cautious interpretation for future longitudinal or interventional studies.

## 11 Future directions

Future obesity microbiome research should move beyond simple comparisons between individuals with obesity and lean or non-obese controls. Although such comparisons remain useful for identifying broad obesity-associated microbial differences, they may obscure clinically relevant heterogeneity within the obesity population. A more informative strategy is to characterize baseline microbiota patterns together with comprehensive assessment of the biological and contextual domains defined in this review. This approach may help determine whether apparently obesity-associated microbial signals remain robust across contextual variation or instead reflect confounding, effect modification, or specific metabolic subphenotypes (57, 58, 60).

Longitudinal studies are needed to determine whether baseline microbiota features are associated with subsequent changes in body weight, adiposity, insulin resistance, liver fat, inflammatory markers, and long-term cardiometabolic outcomes after intervention. Repeated stool sampling before, during, and after dietary, lifestyle, pharmacological, or surgical weight-loss interventions would help distinguish relatively stable baseline microbial traits from dynamic microbiota changes induced by treatment, altered diet, weight loss, or metabolic improvement (21). Such studies should also include standardized clinical endpoints, dietary adherence assessment, medication records, and adequate follow-up duration, because microbiota changes may reflect both intervention exposure and host metabolic response.

Future research should also strengthen causal interpretation. Most human obesity microbiome studies remain observational, and cross-sectional associations cannot determine whether microbial alterations contribute to metabolic dysfunction, result from obesity-related host or dietary changes, or coexist with shared confounders. Controlled preclinical models can help test candidate mechanisms by manipulating microbial communities, defined taxa, microbial metabolites, diet, and host background. However, animal models should be used as mechanistic bridges rather than direct surrogates for human obesity because host physiology, microbial ecology, dietary exposure, and microbial engraftment differ between experimental animals and free-living human populations (25, 80). The strongest causal interpretation will therefore require triangulation across animal experiments, prospective and repeated-measure human studies, controlled interventions, causal inference approaches, and validation in independent populations. Microbial features associated with metabolic status or weight-loss response should remain candidate markers or mechanistic hypotheses unless supported by convergent experimental and human evidence.

Multi-omics approaches may further improve mechanistic interpretation. Although 16S rRNA sequencing is suitable for first-stage taxonomic profiling, integration with shotgun metagenomics, metatranscriptomics, metabolomics, bile acid profiling, short-chain fatty acid measurement, inflammatory markers, and detailed dietary assessment could clarify whether observed microbial patterns are linked to functional pathways relevant to obesity (41, 81). Future studies should also incorporate absolute or microbial-load-aware quantification when feasible, particularly when taxon-level expansion or depletion is central to the hypothesis. Combining sequencing with flow cytometric cell counts, quantitative PCR, or validated internal-standard approaches can help distinguish true changes in microbial quantity from proportional shifts inherent to relative-abundance data (75, 76). Such approaches may help identify microbial functions related to carbohydrate metabolism, bile acid signaling, gut barrier function, inflammatory activation, and host insulin sensitivity. However, multi-omics studies require larger sample sizes, standardized protocols, careful batch control, and appropriate statistical methods to avoid overinterpretation of high-dimensional data.

Future research should also extend baseline profiling beyond the predominantly bacterial communities emphasized in this review. The intestinal ecosystem includes fungal and viral communities, and emerging evidence links variation in the gut mycobiome and virome to obesity while also indicating sensitivity to diet and geographic context (7–9). Future studies should determine whether integrating bacterial, fungal, viral, metabolic, and host data improves characterization of pre-intervention heterogeneity and its relevance to subsequent intervention response.

For underrepresented populations with distinctive dietary and behavioral backgrounds, the immediate priority should be to establish high-quality baseline microbiota datasets in well-characterized obesity cohorts. These studies should be realistic in scope, methodologically standardized, and cautious in interpretation. Their value lies not in proving regional uniqueness at the first stage, but in providing population-specific reference data, identifying major sources of baseline heterogeneity, and generating hypotheses for future mechanistic research (37, 38).

Finally, clinical translation should proceed cautiously. Baseline microbiota profiling may eventually contribute to metabolic risk stratification or personalized intervention selection, but current evidence is not sufficient to support routine microbiota-based prediction of weight-loss response. Future studies should prioritize external validation, reproducibility, clinically interpretable endpoints, and integration with conventional risk factors before proposing microbiota-based clinical decision-making. In this context, pre-intervention microbiota research should be viewed as a foundation for precision obesity research rather than as an immediately actionable diagnostic or predictive tool.

## 12 Conclusion

The central conclusion of this review is not that obesity lacks meaningful microbiome associations, but that these associations cannot be interpreted independently of the baseline context in which they arise or the methodological procedures through which they are observed. Inconsistency across studies should therefore not be regarded solely as failed replication. It may reflect variation across the seven biological and contextual domains defined in this review, as well as differences in cohort definition, sample processing, bioinformatic reconstruction, and statistical analysis.

The proposed baseline heterogeneity framework captures this interpretation through three linked principles: obesity-associated microbiota signals are context-dependent, observed profiles are method- and pipeline-dependent, and their interpretation must be temporally anchored to the pre-intervention state. Unlike taxa-centered syntheses, the framework explains inconsistency by separating interacting biological and contextual determinants from the methodological reconstruction of the observed microbiota profile. Baseline profiling should therefore be viewed not as a search for a fixed microbial definition of obesity, but as the systematic characterization of the biological, environmental, clinical, and methodological conditions that shape an observed microbiota profile.

This conceptual shift has direct implications for future research. Well-characterized baseline cohorts, particularly in underrepresented populations, can identify sources of confounding and effect modification, improve cross-population interpretation, and establish the microbial context from which dietary, lifestyle, pharmacological, or surgical intervention begins. Longitudinal follow-up, independent validation, harmonized methodological reporting, and integration with functional and metabolic measurements will then be required before baseline microbial features can support causal or predictive inference. By shifting the field from asking which microorganisms define obesity to asking why microbiome findings diverge, pre-intervention profiling can provide a more rigorous foundation for reproducible and personalized obesity microbiome research.
